# Mapping Acupoint Networks for Chronic Pelvic Pain: A Review‐Based Network Analysis of Randomized Trials

**DOI:** 10.1155/prm/5799642

**Published:** 2026-06-16

**Authors:** Yewon Baeg, Yi-Chuan Chang, Cheng-Hao Tu, Younbyoung Chae

**Affiliations:** ^1^ Department of Meridian and Acupoints, College of Korean Medicine, Kyung Hee University, Seoul, South Korea, khu.ac.kr; ^2^ Department of Chinese Medicine, Beigang Hospital, China Medical University, Beigang, Taiwan, cmu.edu.cn; ^3^ School of Post-Baccalaureate Chinese Medicine, China Medical University, Taichung, Taiwan, cmu.edu.cn; ^4^ Graduate Institute of Acupuncture Science, China Medical University, Taichung, Taiwan, cmu.edu.tw

**Keywords:** acupuncture, chronic pelvic pain, data mining, network analysis, sex differences

## Abstract

**Background:**

Chronic pelvic pain (CPP) is a heterogeneous clinical condition affecting both women and men, but acupoint selection patterns across CPP‐related randomized trials have not been systematically characterized.

**Objective:**

To identify common acupoint networks used for CPP and to examine sex‐stratified differences in acupoint co‐occurrence patterns.

**Methods:**

This study was a review‐based secondary network analysis of randomized controlled trials included in a previously published systematic review and meta‐analysis of acupuncture for CPP‐related conditions. Among 17 eligible trials identified in the source review, 15 provided sufficient acupoint prescription information for network reconstruction and were included in the present analysis. The dataset comprised 8 female studies and 7 male studies. Acupoint co‐occurrence networks were constructed, and eigenvector centrality was used to identify major acupoints and compare overall and sex‐stratified network structures.

**Results:**

In the overall network, BL32, LI4, CV4, GV20, and BL33 showed the highest eigenvector centrality values. Spatial mapping demonstrated a multiregional configuration integrating abdominal, lumbosacral, and distal limb acupoints. In female and male CPP networks, LI4 and lumbosacral back‐region acupoints such as BL32, BL25, and BL33 were more central in females, whereas CV4, CV3, and lower abdominal acupoints were more central in males.

**Conclusion:**

This review‐based network analysis identified both common and sex‐stratified acupoint prescription patterns for CPP and visualized their anatomical distribution. These findings provide a structured overview of acupoint network organization in CPP and support future outcome‐oriented and disease‐specific acupuncture research.

## 1. Introduction

Chronic pelvic pain (CPP) is commonly defined as persistent or recurrent pain arising from the pelvic region and lasting for more than six months and is often accompanied by cognitive, behavioral, sexual, and emotional disturbances, as well as urinary, bowel, myofascial, or gynecologic symptoms [1, 2]. Because of heterogeneity in case definitions and study populations, the prevalence of CPP is difficult to estimate; however, systematic reviews have reported prevalence ranges of 5.7%–26.6% and 4.0%–43.4% among women [3, 4]. CPP arises from diverse etiologies, including gynecological, gastrointestinal, urological, musculoskeletal, neuropathic, and psychosocial conditions, while men with chronic prostatitis/chronic pelvic pain syndrome (CP/CPPS) experience overlapping urogenital, urinary, sexual, and psychosocial symptoms [5, 6]. Current management of CPP typically relies on a multimodal, interdisciplinary approach that combines pharmacologic and nonpharmacologic interventions. Nevertheless, the evidence supporting many of these treatments remains limited or inconsistent, and some interventions are associated with adverse effects, posing substantial challenges for long‐term management [7].

Acupuncture has been used for centuries in East Asia and has more recently attracted increasing attention as a potential therapeutic option for CPP [8]. Systematic reviews and randomized controlled trials suggest that acupuncture can reduce pain intensity and improve symptom severity in CPP and related conditions, including CP/CPPS and endometriosis‐related CPP [9–12]. Although a recent systematic review of nonpharmacological conservative therapies reported inconsistent conclusions regarding the certainty of evidence for acupuncture, these data nonetheless highlight its potential role as a complementary modality within a multimodal management strategy for CPP [13]. Despite growing evidence supporting the therapeutic effects of acupuncture for CPP, relatively little attention has been paid to the selection patterns of acupoints used in clinical practice. Therefore, it is important to characterize which acupoints are most frequently selected for CPP, how these acupoints are spatially distributed, and how selection patterns differ between female and male patient groups.

Acupoint combinations used in clinical practice can be conceptualized as networks in which individual acupoints are represented as nodes and their co‐occurrence within treatment prescriptions as edges. Network analysis provides a quantitative framework for characterizing such complex systems by representing their components and relationships as graphs [14]. In acupuncture research, network‐based approaches have been applied to identify structural patterns and underlying principles of acupoint selection across diverse clinical conditions. In particular, studies on low back pain, pain‐related disorders, and functional gastrointestinal diseases have demonstrated that acupoint selection follows modular and disease‐specific patterns [15–17]. Taken together, these findings indicate that network analysis is a useful tool for elucidating acupuncture treatment strategies and the specificity of acupoint selection, and they provide a methodological basis for investigating acupoint networks in CPP. However, to date, no study has systematically characterized acupoint prescription structure for CPP using network analysis or compared sex‐stratified prescription patterns across CPP‐related conditions. Although CPP is discussed as a shared clinical pain construct, the underlying disease spectrum differs substantially between female and male populations. Female CPP trials often include conditions such as endometriosis, CPP, and pelvic girdle pain, whereas male CPP trials are largely centered on CP/CPPS. These differences may influence acupoint selection patterns. Therefore, the present sex‐stratified comparison should be interpreted primarily as a comparison of prescription patterns across sex‐associated CPP clinical contexts rather than as direct evidence of biological sex‐specific treatment effects. Within this context, identifying both common and sex‐stratified acupoint network patterns may help distinguish shared treatment cores from clinically contextualized prescribing strategies.

The present study aimed to identify common acupoint combinations used for CPP treatment, characterize their spatial distribution, and examine sex‐stratified differences in acupoint network structure and anatomical patterning across CPP‐related conditions. We extracted acupoint data from randomized clinical trials on endometriosis, pelvic girdle and low back pain, pelvic inflammatory disease, and CP/CPPS included in a previously published systematic review and meta‐analysis, and performed network analysis using centrality measures to visualize acupoint relationships and compare sex‐stratified selection patterns.

## 2. Methods

### 2.1. Data Sources of Acupoints for CPP

This study was a review‐based secondary network analysis of acupoint prescriptions extracted from published randomized controlled trials included in a previously published systematic review and meta‐analysis of acupuncture for CPP‐related conditions [11]. In the source review, randomized controlled trials were searched in PubMed and Embase from January 1, 2011, to September 30, 2022, without language restriction. The search strategy combined CPP‐related terms with acupuncture‐related terms as follows: [(Endometriosis) OR (pelvic pain) OR (chronic pelvic pain) NOT (primary dysmenorrhea)] AND [(Acupuncture) OR (acupressure) OR (electroacupuncture) OR (meridians) OR (moxibustion) OR (needling)]. The source review retrieved 126 records from PubMed and Embase, removed 27 duplicates, screened 99 records by abstract, assessed 48 full‐text reports, and finally included 17 randomized controlled trials for meta‐analysis.

In that review, randomized controlled trials evaluating acupuncture for disorders such as endometriosis‐related CPP, pregnancy‐related pelvic girdle and low back pain, pelvic inflammatory disease, and CP/CPPS were identified. Among 17 eligible randomized controlled trials identified in the source review, 2 were excluded from the present analysis because acupoint prescriptions were not reported in sufficient detail to allow network reconstruction, leaving 15 trials for the final network analysis.

For each included trial, we extracted the clinical condition, target sex (female‐only or male‐only), and the list of acupoints used in the acupuncture intervention arms as reported by the original authors. Trials that included only female participants were assigned to the female‐specific network, whereas trials that included only male participants were assigned to the male‐specific network, resulting in 8 female trials and 7 male trials. The combined dataset from all 15 trials was used to construct the overall CPP acupoint network. The characteristics of the studies included in the present secondary network analysis are summarized in Table 1; the final dataset comprised 8 female studies and 7 male studies.

**TABLE 1:** Summary of the randomized controlled trials included in the present secondary network analysis.

Category	Female studies	Male studies
Number of included RCTs	8	7
Main conditions	Endometriosis, CPP, pelvic girdle pain	CP/CPPS
Main intervention types	EA, MA, moxibustion, ear acupuncture, laser acupuncture	EA, MA, catgut embedding
Main controls	Standard care, medication, physiotherapy	Medication, sham acupuncture

### 2.2. Network Analysis and Visualization

Based on the extracted acupoint data, we constructed three undirected, weighted acupoint networks: an overall CPP network including all 15 trials and sex‐stratified networks including only female or only male trials, respectively. In each network, nodes represented unique acupoints, and edges were defined by the co‐occurrence of two acupoints within the same reported treatment prescription. Edge weights reflected the frequency with which each acupoint pair co‐occurred across the included trials, and these weights were used in the calculation of network measures.

For each acupoint node, we calculated four centrality measures: degree, closeness, betweenness, and eigenvector centrality. Degree represents the number of edges incident to a node, reflecting how many other nodes it directly connects to. Eigenvector centrality quantifies the overall importance of a node based on the centrality of its neighboring nodes, whereas closeness and betweenness centrality indicate, respectively, how efficiently a node can reach other nodes and how frequently it lies on the shortest paths between pairs of nodes [18, 19]. In this study, we primarily used eigenvector centrality to evaluate the relative importance of each acupoint for CPP, because this measure emphasizes acupoints that are connected to other highly central acupoints.

Network construction and visualization were performed in Gephi (Version 0.10.1; https://gephi.org/). For the overall and sex‐stratified networks, we applied the Yifan Hu layout to obtain a two‐dimensional spatial arrangement of nodes. Node size and color were scaled according to eigenvector centrality values, such that acupoints with higher centrality appeared larger and darker, and edge width was scaled according to connection weight. To further explore sex‐stratified spatial patterns, eigenvector centrality values for each acupoint were visualized as a heat map by anatomical region using Orange software (Version 3.28.0; https://orangedatamining.com). By focusing on co‐occurrence structure rather than treatment‐effect synthesis, the present study provides a complementary view of the CPP acupuncture literature based on how acupoints are combined across randomized trials.

## 3. Results

### 3.1. Common Acupoint Combinations for CPP

**FIGURE 1:**
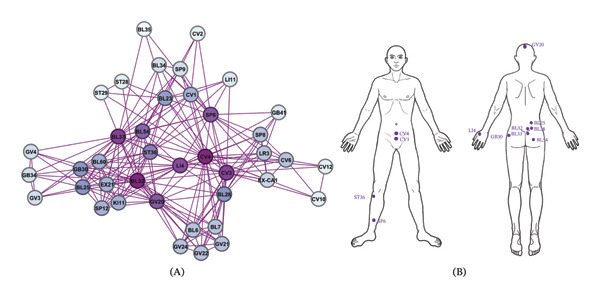
Visualization of common acupoint combinations for chronic pelvic pain. (A) Network of common acupoint combinations for chronic pelvic pain (CPP) (nodes = 40, edges = 218). BL32, LI4, CV4, GV20, and BL33 showed the highest eigenvector centrality values and were therefore identified as major acupoints for CPP treatment. Nodes are colored on a purple scale according to their eigenvector centrality values (darker color indicates higher centrality), and edge width reflects connection weight. Network analysis was performed in Gephi (Version 0.10.1; https://gephi.org/) using the Yifan Hu layout. (B) Anterior and posterior body maps showing the 12 acupoints with the highest eigenvector centrality values and their anatomical locations. Characteristic spatial patterns of acupoint combinations for CPP are observed in the abdominal (CV4, CV3), back (BL32, BL33, BL54, BL28, BL25, GB30), and limb regions (LI4, ST36, SP6).

Network analysis of the overall CPP dataset yielded a co‐occurrence network comprising 40 acupoints and 218 edges, illustrating how acupoints are combined within clinical trial prescriptions (Figure 1A). Across all participants, BL32 showed the highest eigenvector centrality value, followed by LI4, CV4, GV20, and BL33, indicating that these acupoints function as major hubs within the CPP treatment network (Table 2). The 12 acupoints with the highest eigenvector centrality values (BL32, LI4, CV4, GV20, BL33, BL54, CV3, ST36, SP6, BL25, GB30, and BL28) all exhibited relatively high degree values (14–25) and closeness centrality values (0.59–0.74), suggesting that they are densely connected to other acupoints and occupy central positions within the network.

**TABLE 2:** Common acupoint combinations for chronic pelvic pain.

Acupoint	Eigenvector centrality	Degree	Closeness centrality	Betweenness centrality
BL32	1.00	25	0.74	0.14
LI4	0.90	20	0.67	0.08
CV4	0.89	25	0.74	0.16
GV20	0.88	19	0.66	0.04
BL33	0.87	21	0.68	0.09
BL54	0.81	18	0.65	0.04
CV3	0.78	21	0.68	0.09
ST36	0.77	16	0.63	0.02
SP6	0.68	19	0.66	0.07
BL25	0.64	14	0.59	0.01
GB30	0.64	14	0.59	0.01
BL28	0.59	14	0.61	0.02

When these 12 acupoints were mapped onto body diagrams, spatial patterns were found in the abdominal, back, and limb regions (Figure 1B). Abdominal acupoints CV4 and CV3, back‐region acupoints BL32, BL33, BL54, BL28, and BL25, and limb acupoints LI4, ST36, SP6, and GB30 formed a multiregional configuration that links local points near the pelvis with distal extremity points. This pattern suggests that acupuncture prescriptions used in clinical trials of CPP commonly integrate lower abdominal *front-mu*–related points, lumbosacral *back-shu*–related points, and distal limb points, rather than relying on a single anatomical region.

### 3.2. Sex‐Stratified Acupoint Combinations for CPP

**FIGURE 2:**
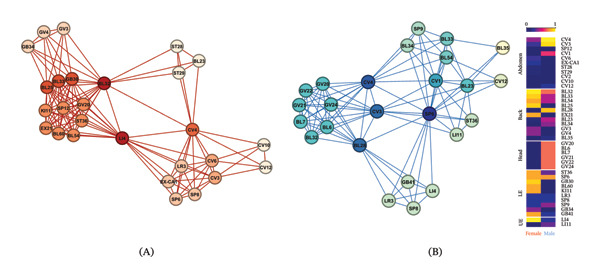
Network analysis of sex‐stratified acupoint combinations for chronic pelvic pain. (A) Network of female‐specific acupoint combinations for chronic pelvic pain (CPP) (nodes = 27, edges = 126). LI4, BL32, BL25, BL33, and GB30 exhibited the highest eigenvector centrality values. Nodes are colored on a red–orange scale according to their eigenvector centrality values (darker color indicates higher centrality), and edge width reflects connection weight. (B) Network of male‐specific acupoint combinations for CPP (nodes = 25, edges = 104). CV4, CV3, BL28, and SP6 exhibited the highest eigenvector centrality values. Nodes are colored on a blue–green scale according to their eigenvector centrality values, and edge width reflects connection weight. In both panels, network analysis was performed in Gephi (Version 0.10.1; https://gephi.org/) using the Yifan Hu layout. Heat map of sex‐stratified spatial patterns in acupoint combinations for CPP, showing normalized eigenvector centrality values for each acupoint by anatomical region (abdomen, back, head, lower extremity, upper extremity) in female and male networks. Warmer colors indicate higher eigenvector centrality values. In females, lumbosacral back‐region acupoints such as BL32, BL25, and BL33 show higher centrality, whereas in males, abdominal acupoints, including CV4 and CV3, are more central. The heat map was generated using Orange software (Version 3.28.0; https://orangedatamining.com).

In female and male CPP network, the female‐specific network consisted of 27 acupoints and 126 edges, whereas the male‐specific network consisted of 25 acupoints and 104 edges (Figure 2A,B; Table 2). In the female network, LI4, BL32, BL25, BL33, and GB30 exhibited the highest eigenvector centrality values, indicating that these acupoints function as major hubs in treatment combinations for female CPP (Table 3). LI4 and BL32 also showed relatively high closeness and betweenness centrality, suggesting that they not only connect to many other acupoints but also lie on key shortest paths linking different parts of the network. In the male network, CV4, CV3, BL28, and SP6 were the most central acupoints by eigenvector centrality, with CV4 and CV3 forming a strongly connected core in the lower abdominal region and SP6 acting as an important bridging point with high betweenness centrality.

**TABLE 3:** Sex‐stratified acupoint combinations for chronic pelvic pain.

Acupoint	Eigenvector centrality	Degree	Closeness centrality	Betweenness centrality
*Female*				
LI4	1.00	18	0.76	0.30
BL32	0.99	18	0.76	0.22
BL25	0.91	14	0.65	0.03
BL33	0.91	14	0.65	0.03
GB30	0.91	14	0.65	0.03
BL54	0.82	11	0.6	0
BL60	0.82	11	0.6	0
EX21	0.82	11	0.6	0
GV20	0.82	11	0.6	0
KI11	0.82	11	0.6	0
SP12	0.82	11	0.6	0
ST36	0.82	11	0.6	0

*Male*				
CV4	1.00	15	0.73	0.16
CV3	0.95	14	0.71	0.13
BL28	0.88	14	0.71	0.13
SP6	0.80	17	0.77	0.25
BL32	0.71	9	0.59	0
BL6	0.71	9	0.59	0
BL7	0.71	9	0.59	0
GV20	0.71	9	0.59	0
GV21	0.71	9	0.59	0
GV22	0.71	9	0.59	0
GV24	0.71	9	0.59	0
CV1	0.60	11	0.65	0.04

Heat map visualization of normalized eigenvector centrality values by anatomical region further revealed these sex‐stratified patterns. In females, back‐region acupoints such as BL32, BL25, and BL33 displayed higher centrality than their abdominal counterparts, indicating a predominance of lumbosacral stimulation. In contrast, in males, abdominal acupoints including CV4 and CV3 were more central than back‐region points, while SP6 and other lower limb acupoints showed prominent contributions. Together, these findings show distinct prescribing patterns across female‐ and male‐stratified trial datasets, with female prescriptions emphasizing lumbosacral *back-shu*–related points and male prescriptions prioritizing lower abdominal and lower limb points.

## 4. Discussion

In this study, we used network analysis to characterize acupoint prescription patterns in clinical trials of acupuncture for CPP. By constructing acupoint networks from 15 randomized controlled trials on CPP‐related conditions and evaluating multiple centrality measures, we identified key acupoints and their spatial distribution, as well as sex‐stratified patterns. In the overall CPP network, BL32, LI4, CV4, GV20, and BL33 showed the highest eigenvector centrality values, indicating that these acupoints function as major hubs within commonly used treatment combinations. Spatial mapping of the most central acupoints revealed a characteristic multiregional prescription pattern integrating abdominal (CV4, CV3), lumbosacral back (BL32, BL33, BL54), and limb (LI4, ST36) acupoints.

Our network analysis identified a small set of highly connected acupoints that serve as hubs in CPP treatment prescriptions, most notably BL32, LI4, CV4, GV20, and BL33. The predominance of abdominal and back acupoints in CPP treatment may be understood in relation to traditional concepts of *front-mu* and *back-shu* points [20]. CV4 and CV3, located in the lower abdomen, are classical *front-mu* points associated with the small intestine and bladder, respectively, and are frequently indicated for lower abdominal and pelvic disorders. Similarly, BL32, BL33, BL25, BL28, and BL54 are located along the Bladder meridian in the lumbosacral region and include *back-shu* points related to pelvic organs. The high eigenvector centrality values observed for these acupoints suggest that combinations involving *front-mu* and *back-shu* points remain prominent in clinical trials of acupuncture for CPP. Moreover, needling of lumbosacral back‐region acupoints such as BL32 and BL33 may exert therapeutic effects by directly stimulating the sacral plexus and modulating nociceptive input from the pelvic organs, which is consistent with neuroanatomical explanations proposed in previous acupuncture research [21, 22].

Our sex‐stratified analyses further revealed distinct prescription patterns between female and male CPP populations. In female‐specific networks, LI4, BL32, BL25, BL33, and GB30 were the most central acupoints, and back‐region acupoints showed higher eigenvector centrality values, indicating a strong emphasis on lumbosacral stimulation. In contrast, male‐specific networks were dominated by CV4, CV3, BL28, and SP6, and abdominal acupoints were more central overall. These differences may reflect underlying sex‐specific disease profiles, such as endometriosis, dysmenorrhea, and pregnancy‐related pelvic girdle pain in females versus CP/CPP syndrome in males, which may lead clinicians to prioritize different anatomical regions and meridian systems. In addition, sex‐related differences in body accessibility and patient preferences may also contribute to these patterns. However, these possibilities remain speculative and should be examined directly in future studies. The observed differences between female and male networks should not be interpreted solely as biological sex effects, because the included studies also differed in clinical diagnoses, treatment contexts, and acupuncture traditions. In particular, female trials mainly involved gynecologic CPP‐related conditions, whereas male trials were largely focused on CP/CPPS, which may have substantially influenced acupoint selection patterns. The identification of such sex‐stratified patterns underscores the potential value of considering predominant clinical phenotype, body‐region acceptability, and sex‐associated treatment contexts when designing CPP acupuncture prescriptions, rather than applying a uniform treatment protocol.

From a clinical perspective, our findings may assist practitioners in designing evidence‐informed acupoint combinations for CPP. The overall network identifies BL32, LI4, CV4, GV20, and BL33 as major hubs that frequently co‐occur with other acupoints, suggesting that these points may serve as a commonly used core set within CPP‐related acupuncture prescriptions. The spatial patterns also support the practice of combining local acupoints in the abdominal and lumbosacral regions with distal limb points such as LI4 and ST36 to achieve both segmental and systemic modulation [15, 23]. In addition, the observed sex‐stratified differences suggest that emphasizing back‐region acupoints in female patients and abdominal‐region acupoints in male patients may better align with prevailing clinical practice patterns and potentially enhance treatment relevance. However, it is important to note that network centrality does not directly equate to clinical efficacy, and the optimal combination of acupoints for individual patients still needs to be validated in prospective clinical trials.

Another notable feature of our network is the high eigenvector centrality of GV20, which suggests a meaningful role for this point in CPP treatment beyond its local effects on the scalp. GV20, located at the vertex along the Governor vessel, is traditionally indicated for headache, dizziness, and psychiatric symptoms, and is often regarded as a key point for regulating “mental” and central nervous system functions [15, 24, 25]. Experimental studies have shown that stimulation of GV20 can modulate cortical and subcortical activity, including regions involved in pain perception, affective processing, and descending pain inhibition, and can influence autonomic outflow and stress‐related responses [26–29]. In the context of CPP—a condition characterized not only by nociceptive input from pelvic organs but also by substantial emotional distress and central sensitization—the frequent co‐use of GV20 with abdominal and lumbosacral acupoints may therefore reflect an integrated strategy that combines segmental modulation of pelvic afferents with supraspinal regulation of pain and mood [30]. The central hub position of GV20 in our network supports the notion that targeting higher order pain modulatory systems is a common and potentially important component of acupuncture prescriptions for CPP.

Several limitations of this study should be acknowledged. First, the acupoint data were derived from a single systematic review and meta‐analysis, and only 15 trials were included after excluding studies with insufficient reporting of acupoint prescriptions, which may limit the generalizability of our findings. Second, CPP encompasses a heterogeneous group of conditions, and we pooled acupoint combinations across gynecologic, urologic, musculoskeletal, and neuropathic etiologies, which may obscure disease‐specific patterns. Third, we focused on acupoint co‐occurrence and centrality metrics instead of treatment outcomes; therefore, we could not examine whether specific acupoint combinations are causally related to effectiveness. Fourth, details such as needling depth, stimulation techniques, retention time, and treatment frequency were not incorporated into the network models, even though these parameters may influence treatment response. Because the present network reconstruction relied on published acupoint prescriptions, insufficient reporting of intervention details may have affected the completeness and reproducibility of the network structure. Finally, variability in methodological quality and clinical heterogeneity across the original trials may also have affected acupoint selection; however, this study was designed primarily to characterize the connectivity pattern rather than to compare treatment efficacy. Future studies should further evaluate the robustness of network structures using sensitivity analyses across different prescription‐selection criteria and reporting quality thresholds.

Despite these limitations, this study demonstrates the usefulness of network analysis as a methodological approach for summarizing and visualizing complex prescribing patterns in CPP. By quantifying the relative importance of acupoints and revealing both general and sex‐stratified spatial patterns, our findings provide a structured overview of how acupuncture is currently applied in CPP‐related clinical trials. Future research should expand the acupoint database to include a broader range of conditions and study designs, perform disease‐specific network analyses, and integrate outcome data to examine whether highly central acupoints or combinations are associated with superior clinical effects. Future outcome‐oriented studies may further examine whether network‐derived acupoint combinations are associated not only with statistical improvement but also with clinically important differences in patient‐reported outcome measures such as the NIH‐CPSI. Prospective trials that compare network‐derived acupoint prescriptions with conventional expert‐based prescriptions would further clarify the clinical utility of this approach. Ultimately, integrating network analysis with traditional meridian theory and modern neurophysiological insights may contribute to the development of more structured and context‐sensitive acupuncture strategies for CPP.

## 5. Conclusion

Using network analysis of acupoint combinations reported in clinical trials of CPP, we identified BL32, LI4, CV4, GV20, and BL33 as key acupoints that form the core of CPP treatment networks. Spatial mapping revealed characteristic patterns across abdominal (CV4, CV3), lumbosacral back (BL32, BL33, BL54), and limb (LI4, ST36) regions, and sex‐stratified analyses showed that back‐region acupoints were more central in females, whereas abdominal acupoints were more central in males. These findings provide a quantitative overview of common and sex‐stratified acupoint combinations for CPP and may help inform future context‐sensitive acupuncture research and prescribing strategies.

## Author Contributions

Cheng‐Hao Tu and Yi‐Chuan Chang conceived the study. Yewon Baeg and Yi‐Chuan Chang analyzed the data. Yewon Baeg, Cheng‐Hao Tu, and Younbyoung Chae drafted the manuscript. Yewon Baeg and Yi‐Chuan Chang contributed equally to this work as co‐first authors.

## Funding

This work was supported by the National Research Foundation of Korea (NRF) grant funded by the Korea government (MSIT) (RS‐2024‐00449485) and Korea Institute of Oriental Medicine (KSN2511011).

## Disclosure

All authors have read and agreed to the published version of the manuscript.

## Ethics Statement

The authors have nothing to report.

## Conflicts of Interest

The authors declare no conflicts of interest.

## Data Availability

The datasets generated and analyzed during the current study are available from the corresponding authors upon reasonable request.
